# Use of Cellular Decapping Activators by Positive-Strand RNA Viruses

**DOI:** 10.3390/v8120340

**Published:** 2016-12-21

**Authors:** Jennifer Jungfleisch, Bernat Blasco-Moreno, Juana Díez

**Affiliations:** Molecular Virology Laboratory, Department of Experimental and Health Sciences, Universitat Pompeu Fabra, Barcelona 08003, Spain; Jennifer.Jungfleisch@upf.edu (J.J.); Bernat.Blasco@upf.edu (B.B.-M.)

**Keywords:** mRNA decay, positive strand RNA viruses, virus–host interactions

## Abstract

Positive-strand RNA viruses have evolved multiple strategies to not only circumvent the hostile decay machinery but to trick it into being a priceless collaborator supporting viral RNA translation and replication. In this review, we describe the versatile interaction of positive-strand RNA viruses and the 5′-3′ mRNA decay machinery with a focus on the viral subversion of decapping activators. This highly conserved viral trickery is exemplified with the plant Brome mosaic virus, the animal Flock house virus and the human hepatitis C virus.

## 1. Introduction

Viruses maintain a constant duel with their infected host cells. Not only do they evolve strategies to circumvent deleterious cellular responses, but they also take advantage of the rich pools of host factors as controllable resources. Hijacking these resources is essential for the expansion of viruses as their gene-coding capacity is limited. One exquisite example of such viral manipulation is the use of the cellular mRNA decay machinery by a group of positive-strand RNA ((+)RNA) viruses. They use different strategies to turn the mRNA decay proteins into allies that support their replication and expansion.

The (+)RNA virus group includes numerous plant, animal and human pathogens such as the hepatitis C virus (HCV) and the emerging mosquito-borne Zika virus (ZIKV), Dengue virus (DENV), West Nile virus (WNV) and Chikungunya virus (CHIKV). Despite their diversity in terms of genome organization or virion morphology, the replication cycle of (+)RNA viruses is highly conserved [1]. Upon entering the target cell and gaining access to the cytoplasm, their single-stranded RNA genomes act like mRNAs and are directly translated by the host translation machinery to express the viral proteins. Once viral proteins accumulate, translation is repressed and the viral genomes are specifically recruited from the cellular translation machinery into membrane-bound viral replication complexes, where they act as templates for replication. Thus, to ensure productive infection, (+)RNA genomes must express enough viral protein to initiate the replication process and must keep their 5′ and 3′ ends intact to synthesize functional copies of the viral RNA. Hence, it is no surprise that for (+)RNA viruses to expand, the host mRNA decay machinery must be tricked (reviewed in [2]).

**Figure 1:**
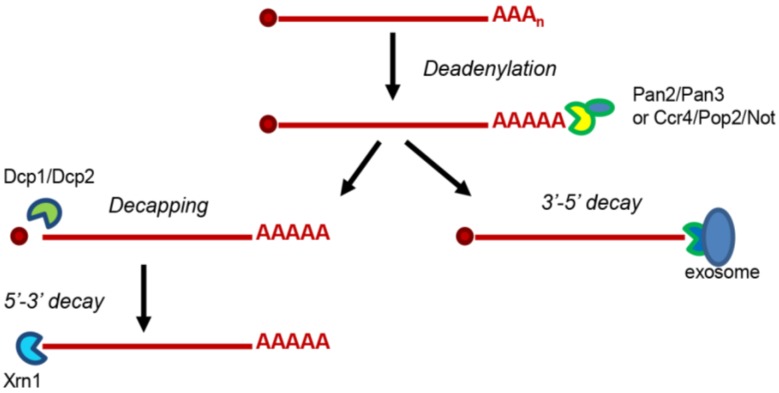
The two main mRNA decay pathways in the cytoplasm: The deadenylation-dependent 5′-3′ decay pathway and the exonucleolytic 3′-5′ decay pathway. Pan2/Pan3: PAB-dependent poly(A)-specific ribonuclease subunits; Pop: PGK promoter directed OverProduction; Not: Negative regulator of transcription subunit; Ccr4: C-C motif chemokine receptor 4; Dcp1/Dcp2: mRNA-decapping enzyme subunit 1/2; Xrn1: 5′-3′ exoribonuclease 1.

Cytoplasmic mRNA decay occurs via two major pathways—the deadenylation-dependent 5′-3′ decay and the exonucleolytic 3′-5′ decay pathways—that are conserved in all eukaryotes (Figure 1) [3]. In both pathways, the 3′ poly(A) tail protects the mRNAs from degradation. Consequently, mRNAs are targeted to degradation only after the deadenylation complex Ccr4/Pop2/Not or Pan2/Pan3 [4,5] shortens the 3′ poly(A)-tail. This leads to the opening of the closed-loop messenger ribonucleoprotein particle (mRNP) formed between the poly(A)-binding protein (PABP) and the cap-complex. Deadenylation is modulated by translation per se, by RNA binding proteins or by stress. Once deadenylation has occurred, mRNAs undergo degradation in the 5′-3′ direction, via decapping and subsequent degradation, or/and in the 3′-5′ direction, via the exosome complex. Besides these two major mRNA decay pathways, there are several specialized ones that primarily function in response to aberrancies in translation and are hence called mRNA quality control pathways (reviewed in [6]). These pathways are based on either deadenylation-independent decay [7], rapid 3′ to 5′ decay [8] or endonuclease cleavage [9] and include the nonsense-mediated decay (NMD), the no-go decay (NGD) and the non-stop decay (NSD) pathway.

The deadenylation-dependent 5′-3′ decay pathway is the main cytoplasmic decay pathway (reviewed in [3]). mRNA-decapping enzyme subunit 2 (Dcp2) and 5'-3' exoribonuclease 1 (Xrn1) are the two key enzymes in this pathway. Dcp2 cleaves the cap structure at the 5′ end of the mRNA, releasing a 7-methylguanosine diphosphate (m^7^GDP) and a 5′ monophosphate mRNA. To be fully active, Dcp2 requires a conformational change mediated by the Dcp1 protein. The other enzyme is the 5′-3′ exonuclease Xrn1 that degrades the mRNA after decapping. In addition, there are other factors, named decapping activators, that assist and enhance the efficacy of the pathway. They include Sm-like proteins 1–7 (Lsm1–7), DNA topoisomerase 2-associated protein (Pat1), DExD/H-box ATP-dependent RNA helicase 1 (Dhh1), enhancer of mRNA-decapping protein 1–3 (Edc1–3) and the Suppressor of Clathrin Deficiency (Scd6). From these, Lsm1–7, Pat1 and Dhh1 are the most characterized ones. The Lsm1–7 ring is constituted by seven Lsm proteins that belong to the conserved Sm family of proteins and acts as an RNA chaperone facilitating a variety of RNA-RNA and RNA-protein interactions [10]. Pat1 functions as a scaffold protein, allowing the sequential binding of decay factors on mRNPs that eventually leads to degradation [11]. Lsm1–7 and Pat1 are co-purified from yeast extracts as a complex [12]. Finally, Dhh1 belongs to the family of DEAD box helicases characterized by acting as RNA chaperones as well. In the 5′-3′ deadenylation-dependent decay pathway, degradation occurs in three-steps. First, translation initiation is inhibited, as mRNA translation initiation and mRNA decay are connected processes in dynamic competition. This is achieved by the concerted action of the deadenylation complex and the decapping activators Dhh1, Lsm1–7, Pat1 and Scd6. Deadenylation leaves the 5′ cap structure accessible for the decapping complex Dcp1/Dcp2 while Dhh1, Pat1 and Scd6 inhibit translation initiation [13,14,15]. Dhh1 also hinders translation elongation by slowing down the ribosomes [16]. Although repression of translation initiation is required for decapping, it does not inevitably lead the mRNA to decapping as some mRNAs remain in a translationally-repressed state. Such translationally-repressed mRNAs can be stored in processing bodies (P-bodies), non-membranous dynamic cytoplasmic foci, and go back to translation or be further processed for degradation [17,18]. Second, the 5′ cap is removed by the Dcp1/Dcp2 decapping complex. The activity of the complex is accelerated by the decapping activators Lsm1–7, Edc1–3 and Pat1 [14,19,20,21]. Third, the exonuclease Xrn1 now has access to the uncapped mRNA and degrades it in the 5′-3′ direction.

The (+)RNA viruses have developed a myriad of strategies to shield their RNA from degradation by Xrn1, often by directly suppressing or degrading the cellular decay machinery. For example, picornaviruses use an aggressive mechanism to combat decay by inducing the rapid degradation of Xrn1 and Dcp1 [22,23]. WNV and DENV use Xrn1 to specifically generate sub-genomic flavivirus RNA (sfRNA) from the genomic RNA (gRNA) [24]. Degradation of gRNA by Xrn1 is stopped by a highly conserved RNA structure at the beginning of the 3′ untranslated region (UTR). The generated sfRNA plays essential roles in viral replication and pathogenesis in human hosts [24] and in mosquitoes as it inhibits the RNA interference (RNAi) response [25] and determines the infection and transmission rates [26]. Interestingly, the generated sfRNA displays an additional role. It binds to and inhibits Xrn1, hence, the endogenous mRNA turnover is altered [27,28,29]. This deregulated host mRNA stability is directly related to sfRNA expression and plays an important role in pathogenesis. As found for WNV and DENV, both the HCV and the Bovine viral diarrhea virus (BVDV) contain regions that stall and repress the enzymatic activity of Xrn1 [30]. However, in these two viruses, the regions are located in the 5′ UTRs. Intriguingly, other (+)RNA viruses, rather than avoiding or using the degradation activity of the 5′-3′ deadenylation-dependent decay machinery, redirect it to other functions. This review describes the strategies of the Brome mosaic virus (BMV), the Flock house virus (FHV) and HCV as representative plant, animal and human (+)RNA viruses that subvert the cellular decapping machinery to promote translation and replication of their viral RNA genomes.

## 2. The Brome Mosaic Virus Converts Enemies into Collaborators in Order to Promote Viral RNA Translation and Replication

A fruitful model system to study (+)RNA virus–host interactions is the replication of the plant BMV in *Saccharomyces cerevisiae* (reviewed in [31,32]). The BMV genome consists of three RNAs that are capped at their 5′ end, and at their 3′ end carry a conserved tRNA-like structure (TLS) instead of a poly(A) tail. Both UTRs contain overlapping sequences that control translation and the initiation of negative-strand synthesis (reviewed in [33]). RNA1 and RNA2 encode helicase 1a and polymerase 2a, respectively. The helicase 1a protein is the only viral protein required to recruit the BMV genome from the cellular translation machinery to the viral replication complex. RNA3 encodes the movement protein 3a and through a sub-genomic RNA generated during replication, the coat protein. Both proteins are required for the systemic infection of plants but not for viral replication.

**Figure 2:**
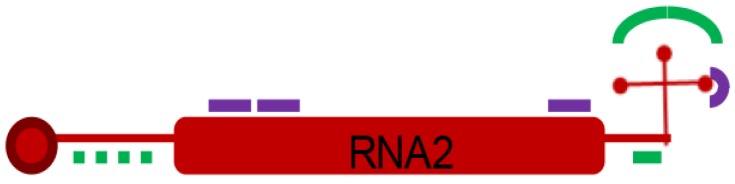
Binding pattern for DExD/H-box ATP-dependent RNA helicase 1 (Dhh1) and the Lsm1–7/Pat1 complex to brome mosaic virus (BMV) RNA2. Dhh1 binds to three sites in the open reading frame (ORF) and to the tRNA-like structure (TLS) in the 3′ untranslated region (UTR) (purple color). The Lsm1–7/Pat1 complex binds to both the TLS and the non-tRNA-like structure in the 3′ UTR and also with lower affinity to the 5′ UTR (green color).

Studies with the BMV/yeast model system led to the identification and characterization of hundreds of host factors required for different steps in the BMV life cycle [34,35]. Three unexpected factors were the decapping activators Lsm1–7, Pat1 and Dhh1. Depletion of the Lsm1–7, Pat1 or Dhh1 proteins dramatically reduced both BMV RNA translation and recruitment from translation to replication of the BMV RNA genomes [36,37,38,39,40,41]. Other components of the decay machinery are not required for these functions, indicating that BMV specifically subverts a selected group of decay factors [39]. The role of the Lsm1–7/Pat1 complex and the RNA helicase Dhh1 in translation has been thoroughly characterized. Both the Lsm1–7/Pat1 complex integrity and its intrinsic RNA-binding activity are required for translation of BMV RNAs [35]. The Lsm1–7/Pat1 complex directly interacts with sequences in both BMV RNA UTRs and with two internal A-rich single-stranded regions located in one of the BMV RNAs [34,35]. These sequences include well-characterized RNA motifs that control BMV RNA translation and replication. In turn, the helicase Dhh1 directly interacts with BMV RNA 3′ UTRs but not with the 5′ UTR. However, Dhh1 interacts with the translation initiation factors eIF4E, eIF4A and eIF4G located at the 5′ UTRs [42]. In addition, Dhh1 was found to bind sequences within the open reading frame (ORF) of BMV RNA2 (Figure 2). Importantly, Lsm1–7/Pat1 and Dhh1 targeted sequences are linked to the dependence on Lsm1–7/Pat1 and Dhh1 for translation, suggesting that their intrinsic RNA binding characteristics determine their function [37,39,42]. Recent exciting results indicate that Dhh1 also promotes translation of a specific set of cellular mRNAs encoding membrane and secreted proteins [42]. Viral and cellular Dhh1-dependent mRNA share some common key features. First, they contain long and highly structured 5′ UTRs and ORFs, including a region located close to the starting AUG. Second, they are directly bound by Dhh1 with a specific binding distribution. Third, they are likely activated by Dhh1 at the translation initiation step. Last, they encode proteins that localize in membranes. Whether Lsm1–7/Pat1 may exert a similar function on cellular mRNAs remains unknown.

**Figure 3:**
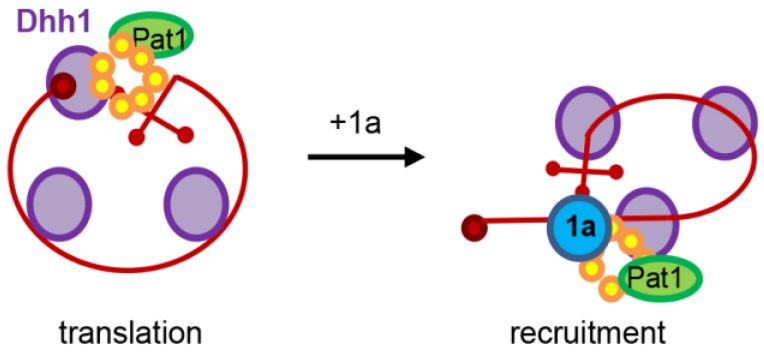
Model of the Lsm1–7/Pat1 complex and Dhh1 function in viral RNA translation and recruitment.

The mechanisms by which the Lsm1–7/Pat1 complex promotes translation and recruitment of BMV RNAs are different. Mutations in the *LSM1* gene, a key component of the Lsm1–7 ring, affect differentially BMV RNA translation and recruitment [35]. Importantly, the Lsm1–7 Pat1 complex interacts in a RNAse-resistent manner with the BMV 1a, the solely viral protein required for recruiment. In line with this, the RNA-biding activity of the Lsm1–7/Pat1 complex is not requried for its function in recruitment [38]. The molecular mechanisms by which Dhh1 promotes BMV RNA recruitment remain uncharacterized. Taken together, the decapping activators Lsm1–7, Pat1 and Dhh1 bind specifically to the BMV RNA genome, promoting its translation and replication rather than its decay. Current results support a model (Figure 3) in which Lsm1–7, Pat1 and Dhh1 bind to *cis*-elements in the viral RNA, thereby remodeling mRNA secondary structures and promoting its circularization and translation. Since poly(A)-tails in cellular mRNAs mediate 5′-3′ circularization via the binding of the poly(A) binding protein, binding of Lsm1–7, Pat1 and Dhh1 to the 5′ and 3′ UTRs structure and to initiation factors at the 5′ UTR would establish such circularization in BMV RNAs. During recruitment, circularization would be disrupted as the Lsm1–7/Pat1 complex would now bind to the viral 1a protein, driving the viral RNA from translation to replication.

## 3. The Flock House Virus Subverts Features of Decapping Proteins to Control the Genomic to Sub-Genomic Viral RNA Ratio

The FHV (reviewed in [43]) is an insect pathogen that can replicate in a wide variety of hosts, including *Drosophila*, *Caenorhabditis elegans*, plants, mammals and *Saccharomyces cerevisiae*. Accordingly, the host factors hijacked by FHV to replicate are highly conserved [44,45,46,47]. The simplicity of the FHV genome, combined with the advantages of yeast genetics make the FHV-yeast system another excellent model system to study basic aspects of (+)RNA biology, including virus–host interactions. Interestingly, three of the highly conserved host factors subverted by FHV are the decapping activators Lsm1–7, Pat1 and Dhh1 [48].

The FHV bipartite genome consists of two capped but non-polyadenylated RNA segments. RNA1 encodes protein A, the only FHV protein required for replication, and RNA2 encodes the capsid precursor. During replication, RNA1 also produces sub-genomic RNA3 that encodes protein B1, of unknown function, and protein B2, required to suppress RNA silencing in infected hosts. RNA3 corresponds to the 3′ end of RNA1 and it is synthesized during RNA1 replication. In addition to its coding function, RNA3 coordinates the production of appropriate levels of RNA1 and RNA2 [49,50,51]. This latter activity is essential for proper and timely expression of the different viral proteins throughout the different stages of the viral life cycle. Interestingly, depletion of Lsm1–7, Pat1 or Dhh1 disrupts this activity and alters the RNA1/RNA3 ratio [48].

Lsm1–7, Pat1 and Dhh1 control RNA3 synthesis [48]. Different RNP rearrangements of the genomic RNA1 are necessary for the viral polymerase to synthesize a complete copy of RNA1 or a partial one, RNA3 [50]. RNA3 synthesis requires a long-distance base pairing interaction between *cis*-elements in RNA1 [50]. These base pairing interactions stop the polymerase prematurely and lead to the synthesis of RNA3 instead of RNA1. As Dhh1 remodels RNP compositions in an ATPase-dependent manner [52] and RNA3 synthesis requires the ATPase activity of the Dhh1 helicase [48], Lsm1–7, Pat1 and Dhh1 have been proposed to regulate the key viral RNP transitions required to maintain the balance between the alternative FHV RNA1 conformations controlling RNA3 synthesis [48]. Interestingly, and as found for BMV, Lsm1–7, Pat1 and Dhh1 interact not only with the RNA genome but also with the viral polymerase [48].

## 4. Subverting Decapping Activators Is Conserved in Human (+)RNA Viruses

Since the 5′-3′ decay pathway is strongly conserved from yeast to humans, human (+)RNA viruses might as well subvert it to favor viral replication. Indeed, the human counterparts of Lsm1–7, Pat1 and Dhh1—namely Lsm1–7, PatL1 and DEAD-Box Helicase 6 (DDX6)—are required for the expansion of the flaviviruses HCV, DENV and WNV. Depletion of Lsm1–7, PatL1 or DDX6 directly and specifically inhibits HCV RNA translation and replication [53,54]. Moreover, in vitro binding assays demonstrated direct interactions of human Lsm1–7 complexes with essential translation/replication regulatory sequences in the 5′ and 3′ UTRs of the HCV RNA genome [53]. DDX6 also interacts with the HCV RNA genome and the core protein in HCV-infected cells [55]. Likewise, DDX6 and Lsm1 promote replication of the WNV and co-localize with the viral replication complex [56]. Moreover, DDX6 directly interacts with structured regulatory *cis*-sequences in the DENV RNA genome and it co-localizes with the DENV replication complex [57].

Although all the above described examples of (+)RNA viruses belong to the *Flaviviridae* family, the conserved use of decapping factors by (+)strand RNA viruses that infect plants (BMV), insects (FHV) and even bacteria (Qβ [58]) underlines the robustness of this strategy to regulate (+)RNA virus life cycles and suggests that it extends to other human viruses outside the *Flaviviridae* family. Depriving (+)RNA viruses of this highly conserved strategy by targeting these decay factors with drugs would therefore appear to be a promising strategy to generate broad-spectrum antiviral drugs. The fact that the individual, transient knock-down of Lsm1–7, PatL1 or DDX6 proteins in human cells is not toxic and that the respective yeast knockout strains are viable, stresses the feasibility of such an approach for the future.

What are the common features of the aforementioned activities of Lsm1–7, Pat1/PatL1 and Dhh1/DDX6 in (+)RNA viral life cycles? In all cases, the decapping activators interact with viral replication proteins, and/or with specific and structured regulatory *cis*-acting signals in the viral RNA genome. The role of these decapping proteins as catalyzers of mRNP transitions that direct cellular mRNAs from translation to decay suggests that they act similarly on the highly structured viral (+)RNA genomes, directing them to translation or replication.

## 5. (+)RNA Viruses Alter the Distribution of Decay Factors

**Figure 4:**
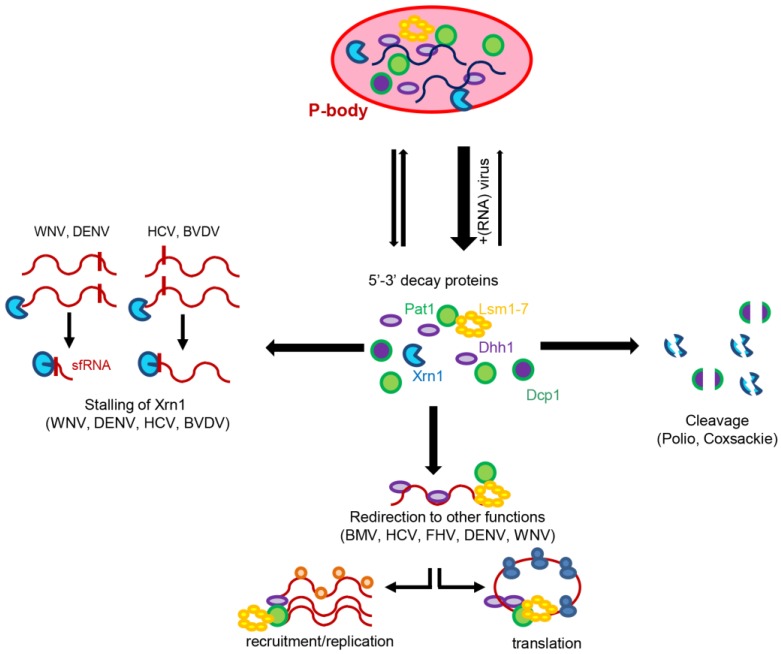
(+)RNA viruses and the cellular mRNA decay machinery. Multiple strategies have been developed by (+)RNA viruses to not only prevent the degradation of the viral RNA genomes but also, to subvert it to their benefit. P-bodies: processing bodies; WNV: West Nile virus; DENV: Dengue virus; HCV: hepatitis C virus; BVDV: bovine viral diarrhea virus; FHV: Flock house virus; BMV: brome mosaic virus.

Lsm1–7, PatL1 and DDX6 accumulate in P-bodies [59,60]. P-bodies are discrete and highly dynamic cytoplasmic mRNP granules found in eukaryotic cells under normal growth condition [59]. These structures contain translationally-repressed mRNAs together with multiple proteins from the 5′-3′ mRNA decay and silencing machineries [60]. Once in P-bodies, mRNAs can be either degraded or stored for future translation [17,61,62]. The components of P-bodies cycle rapidly in and out of these granules, indicating that there is a constant exchange with the cytoplasm where all these components are diffusely distributed [63,64,65]. Importantly, the formation of P-bodies requires most of its components [11,63,66,67,68,69]. Consequently, conditions that reduce the cytosolic concentration of P-body proteins, such as (+)RNA virus infection, disrupt the formation of P-bodies (Figure 4). For example, the picornaviruses poliovirus and Coxsackie virus disrupt P-bodies by degrading the P-body core components Xrn1 and Dcp1a [22,23], and HCV, WNV and DENV do so by preventing Lsm1–7, PatL1 and DDX6 from participating in P-body formation [70,71,72]. All these results were obtained in cell culture infection systems. Importantly, HCV inhibits P-body granule formation in human livers regardless of viral phenotype, inflammation grade or whether infection was recent or long established. Moreover, this alteration is reversed once HCV is eliminated by therapy. Therefore, there is a link between P-body alterations and pathogenic conditions [73].

The obvious question to ask is whether P-body disruption is required for viral infection or whether it is just a consequence. The data currently available suggest that it is a mere consequence, as P-body formation is not required for mRNA decay [74] and depletion of the P-body component Rap55 disrupts P-body formation but it does not affect HCV expansion [70]. Irrespective of the question whether P-body disruption is the consequence or the trigger of pathogenesis, it seems obvious that changing the equilibrium of granulated versus free P-body proteins, proteins that control decay and silencing, would alter the transcriptome and translatome and consequently gene expression. These alterations might be linked to different viral pathologies. Accordingly, to further study the interactions of (+)RNA viruses with P-bodies represents an interesting field that may open many opportunities in terms of therapeutic strategies.

## 6. Concluding Remarks

Viruses are masters in converting hostile conditions into a paradise for their own replication. The sequestering of the cellular decay machinery by (+)RNA viruses is a remarkable example of this that goes beyond using the decay components themselves towards altering the whole transcriptional/translational landscape of the host. The strong conservation of this viral strategy across species pinpoints a weak spot that can be exploited for the development of broad-spectrum antiviral drugs. Yet, some essential questions remain open. For example, how are viral infection, the host transcriptome/translatome and pathogenesis linked? How do viruses regulate host transcription to favor their own replication? The basic nature of these questions highlights that scientists in this area still have a long path to go.
